# Hypoxemia and arousals modulate cardiac responses to respiratory events in obstructive sleep apnea

**DOI:** 10.1093/sleep/zsaf382

**Published:** 2025-12-01

**Authors:** Serajeddin Ebrahimian, Saara Sillanmäki, Marika Rissanen, Eric Staykov, Antti Kulkas, Juha Töyräs, Raquel Bailón, Ludger Grote, Maria R Bonsignore, Mathias Baumert, Virend K Somers, Philip Terrill, Timo Leppänen, Samu Kainulainen

**Affiliations:** Department of Technical Physics, University of Eastern Finland, Kuopio, Finland; Diagnostic Imaging Center, Kuopio University Hospital, Kuopio, Finland; Diagnostic Imaging Center, Kuopio University Hospital, Kuopio, Finland; Institute of Clinical Medicine, University of Eastern Finland, Kuopio, Finland; Department of Technical Physics, University of Eastern Finland, Kuopio, Finland; Diagnostic Imaging Center, Kuopio University Hospital, Kuopio, Finland; School of Electrical Engineering and Computer Science, The University of Queensland, Brisbane, Australia; Department of Technical Physics, University of Eastern Finland, Kuopio, Finland; Department of Clinical Neurophysiology, Seinäjoki Central Hospital, Seinäjoki, Finland; Department of Technical Physics, University of Eastern Finland, Kuopio, Finland; School of Electrical Engineering and Computer Science, The University of Queensland, Brisbane, Australia; Science Service Center, Kuopio University Hospital, Kuopio, Finland; Biomedical Signal Interpretation and Computational Simulation (BSICoS) Group, Aragón Institute of Engineering Research (I3A), IIS Aragón, University of Zaragoza, Zaragoza, Spain; Centro de Investigación Biomédica en Red en Bioingeniería, Biomateriales y Nanomedicina (CIBER-BBN), Madrid, Spain; Centre for Sleep and Vigilance Disorders, Sahlgrenska Academy, Gothenburg University, Gothenburg, Sweden; PROMISE Department, University of Palermo and IFT-CNR, Palermo, Italy; School of Electrical and Mechanical Engineering, The University of Adelaide, Adelaide, Australia; Department of Cardiovascular Medicine, Mayo Clinic, Rochester MN, United States; School of Electrical Engineering and Computer Science, The University of Queensland, Brisbane, Australia; Department of Technical Physics, University of Eastern Finland, Kuopio, Finland; Diagnostic Imaging Center, Kuopio University Hospital, Kuopio, Finland; School of Electrical Engineering and Computer Science, The University of Queensland, Brisbane, Australia; Department of Technical Physics, University of Eastern Finland, Kuopio, Finland; Diagnostic Imaging Center, Kuopio University Hospital, Kuopio, Finland

**Keywords:** obstructive sleep apnea, cardiac response, ventricular repolarization, desaturation, sleep arousal

## Abstract

**Study Objectives:**

Respiratory events during sleep induce immediate cardiac alterations, including increased RR intervals during events and decreased RR intervals after events. However, the extent to which related desaturations and arousals modulate these responses remains underexplored. We hypothesized that desaturations and arousals are the main contributors to respiratory event-related cardiac response, with greatest cardiac alterations expected when both are present.

**Methods:**

We analyzed RR, QT, and heart rate-corrected QT (QTc) intervals before, during, and after 4310 respiratory events from 129 obstructive sleep apnea patients. Mixed-effect statistical models were utilized to assess the influence of the presence and severity of desaturations and arousals on the cardiac electrical response to respiratory events.

**Results:**

There were no significant differences between pre- and post-respiratory event RR and QTc intervals in the absence of desaturation or arousal. Arousal (RR estimate = −23.9 ms; QTc estimate = 5.7 ms) or simultaneous desaturation and arousal (RR estimate = −32.8 ms; QTc estimate = 8.4 ms) modulated significantly (*p* < .05) mean RR and QTc intervals after respiratory events. Desaturation alone affected only RR intervals (estimate = −9.3 ms, p<.05). Greater desaturation depth (RR estimate = −0.09 ms; QTc estimate = 0.56 ms) and longer arousal duration (RR estimate = −3.52 ms; QTc estimate = 0.84 ms) were significant (*p* < .05) predictors of RR and QTc magnitude alterations after respiratory events.

**Conclusions:**

Not all respiratory events have the same effects on cardiac electrophysiology; they are associated with acute alterations after events if they cause desaturations and/or arousal, with longer arousal and deeper desaturations increasing the magnitude of cardiac responses.

**Statement of Significance**

This study provides a detailed analysis of the temporal dynamics of cardiac response to respiratory events in obstructive sleep apnea patients, with a focus on the potential modulators of this response. Our observations present that not all respiratory events have the same effects on cardiac electrophysiology; they are associated with significant cardiac response if they cause desaturations and/or arousals, with longer arousal and deeper desaturations increasing the magnitude of cardiac response. Our results present evidence of possible modulators of post-event cardiac function, providing a deeper understanding of obstructive sleep apnea’s impact on health as potential underlying mechanisms leading to adverse cardiovascular events.

## Graphical Abstract

**Graphical Abstract:**
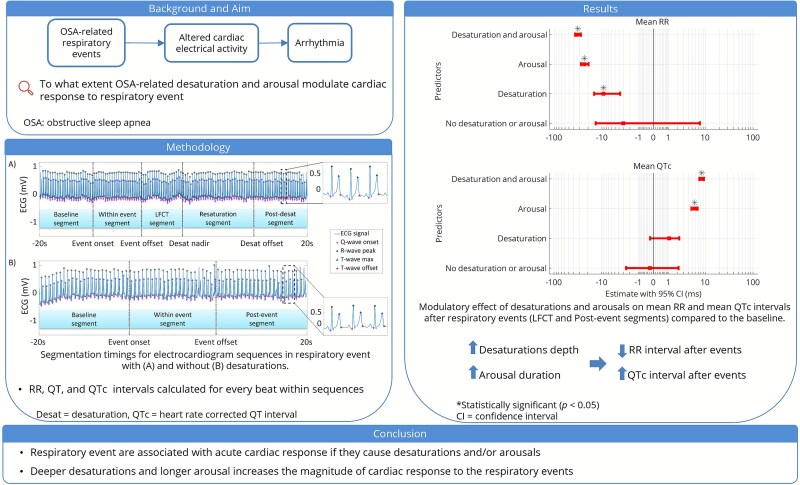

## Introduction

Obstructive sleep apnea (OSA) is a common sleep disorder affecting nearly 1 billion adults worldwide [1]. It is especially prevalent in patients with cardiovascular disease [2] and is a recognized risk factor for sudden cardiac death (SCD) [3]. However, the current clinical OSA severity measure, the apnea-hypopnea index (AHI), is based solely on the frequency of respiratory events and does not consider the immediate impacts of respiratory events (apneas and hypopneas) on cardiovascular function. The current evidence indicates a substantial increase in the relative risk of nocturnal arrhythmia after respiratory event-related disturbances [4], suggesting an immediate effect of respiratory events on cardiac electrophysiology and arrhythmia. Respiratory events elicit alterations in the sympathovagal balance characterized by increased cardiac parasympathetic activity during respiratory events, increased sympathetic activity at respiratory event termination [5], and parasympathetic tone withdrawal upon breathing resumption [6]. OSA-related hypoxemia, arousals, and intrathoracic pressure swings are potential modulators of respiratory event-related changes in autonomic activity [7], which might induce cardiac activity variations reflected by electrocardiogram (ECG) changes. Abnormalities in the cardiac repolarization, such as in heart rate-corrected QT interval (QTc) prolongation, are associated with an increased risk of arrhythmia [8] and SCD [9].

Despite the immediate impact of respiratory events on cardiac function, the temporal dynamics of these variations and the role of possible modulatory mechanisms remain relatively underexplored. Previous studies indicate that there is a pattern of increased RR interval within the respiratory event [10] and decreased RR interval after event termination [11, 12]. However, decreases in post-event RR intervals are more pronounced in association with scored arousals or deep desaturations [12]. In addition, hypoxemia and arousals may independently lead to immediate variations in cardiac electrical activity, demonstrated by decreased RR interval [13, 14], decreased QT interval [1,4], and increased QTc interval [13] and QT variability [15]. However, a comprehensive understanding of the temporal dynamics of OSA-related cardiac electrophysiological alterations is lacking. In particular, nocturnal respiratory events can occur in close succession and thus may either mask or amplify the actual temporal cardiac electrical alterations and lead to over- or under-estimations of cardiac response. It is also unclear how respiratory event-related cardiac electrical activity differs from the periods unaffected by respiratory event-induced alteration, and whether respiratory event-related alterations are independent of possible confounding factors, potential pre-existing cardiac alterations, and patient-specific differences. Additionally, it remains unclear to what extent desaturations, arousals, and sleep stages modulate these variations. Therefore, we sought to explore respiratory event-induced temporal alterations in cardiac electrical activity and identify contributors to the dynamics of alterations. We hypothesized that desaturations and arousals are the main contributors to post-event variations in cardiac electrical activity, with the most prominent cardiac alterations occurring when the respiratory event trigger both phenomena.

## Materials and Methods

### Dataset

This retrospective study was based on a subpopulation of a dataset comprising over 900 type I polysomnography (PSG) recordings of suspected OSA patients conducted with the Compumedics Grael acquisition system (Compumedics, Abbotsford, Australia) at the Princess Alexandra Hospital (Brisbane, Australia) between 2015 and 2017. Of these, 548 patients had medication information available, and of these 134 patients satisfied the following inclusion criteria: more than 4 h of objective sleep, no comorbidities (except diagnosed hypertension) possibly affecting cardiac health (i.e. type 2 diabetes mellitus, chronic obstructive pulmonary disease, hypothyroidism, arrhythmia history, or stroke), no previous cardiac or respiratory failure, no pacemaker, no atrial fibrillation/flutter during PSG, no history of using medication possibly affecting cardiac electrophysiology (i.e. beta-blockers, calcium channel blockers, antipsychotics, or antiarrhythmics), sufficient quality of ECG and nasal airflow signals, and no apparent T-wave abnormalities in ECG signals during PSG (i.e. inverted T-wave, biphasic T-wave, or prominent U-wave). All patients underwent the PSG study due to OSA suspicion and thus were not treated for OSA before or during the PSG recording. The flowchart of inclusion/exclusion criteria is presented in Figure S1. The PSGs were scored manually according to the American Academy of Sleep Medicine 2012 guidelines [16]. A detailed description of the scoring protocol has been published previously [17]. Approval for the collection of retrospective data and its reuse was granted by The Metro South Human Research Ethics Committee, Brisbane, Australia (HREC/16/QPAH/021 and LNR/2019/QMS/54313). All procedures performed in studies involving human participants were done in accordance with the ethical standards of the institutional and/or national research committee and the Declaration of Helsinki.

### Data analysis

ECG signals were recorded with a sampling frequency of 256 Hz using a modified lead II configuration. ECG signals were filtered with a fourth order Butterworth band-pass filter (0.05–40 Hz) before further processing. The nasal airflow signal was utilized to assess the flow-limitation status of patients on a breath-by-breath basis. Each identified breath was classified as normal breathing, possible flow-limited, or certain flow-limited by a fully automated procedure [18, 19].

**Figure 1:**
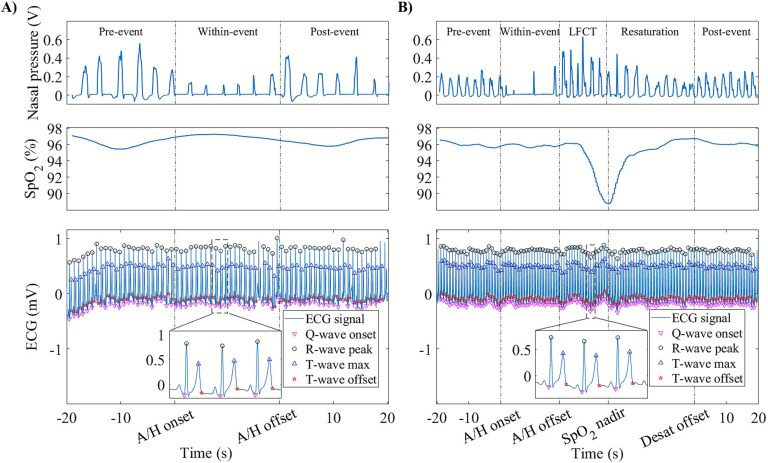
Segmentation process of electrocardiogram (ECG) sequences for (A) respiratory events with no related desaturation and (B) events with related desaturation. The respiratory event was identified from the nasal pressure signal (upper figure), oxygen saturation (SpO_2_) signal was used to detect the presence of a desaturation event ≥3 per cent (middle figure), and ECG sequences were segmented and delineated (bottom figure). A/H = apnea or hypopnea, LFCT = lung-to-finger circulation time, Desat = desaturation.

To capture the dynamics of ventricular repolarization variations due to respiratory events and their immediate consequences, we considered a sequence of segments for each respiratory event, including a 20-s pre-event segment before the onset of the respiratory event, a within-event segment during the entire respiratory event (i.e. from respiratory event onset till respiratory event offset), and a 20-s post-event or post-desaturation segment after the end of the respiratory event (Figure 1, A) or end of related desaturation event (Figure 1, B). In case the respiratory event led to a desaturation, two additional segments were considered: a segment from the respiratory event offset to the nadir of a desaturation event equivalent to lung-to-finger circulation time (LFCT), and a segment from the nadir of a desaturation event to normal oxygenation (resaturation; Figure 1, B). In the case of several overlapping sequences, only the first sequence was included if its post-event or post-desaturation segments did not overlap with the within-event segment of adjacent event; the rest were excluded to avoid misinterpretation of the dynamics of variations in the case of consecutive respiratory events (*n* = 10 438). We further excluded the sequences where the desaturation event started and ended within the respiratory event (*n* = 138) and LFCT was too short (<4 s) or too long (>55 s) to be physiologically plausible (*n* = 283).

ECG sequences were up-sampled to 1000 Hz and automatically delineated using a wavelet-based ECG delineator [20]. The ECG delineations algorithm utilizes the extrema and zero crossings of the signal’s wavelet transform at different scales to delineate wave points within each beat [20]. All sequences were automatically inspected for ectopic beats [21]. Sequences with a mean heart rate <30 bpm and beat delineation rejection >10 per cent (beats could not be delineated due to the high noise and ectopic beat) were excluded from the analysis (*n* = 267). Otherwise, only noisy and ectopic beats in a segment were excluded from the analysis. The final number of analyzable sequences was 4310 (Figure S1). After applying exclusion criteria, 129 patients contributed at least one sequence of segments to the analysis (Table 1). The number of included sequences from each patient is presented in Figure S2. Included sequences were further divided into three groups depending on whether respiratory events were associated with a desaturation ≥3 per cent (*n* = 2378), only arousal (*n* = 1722), or no desaturation or arousal (*n* = 210). The RR interval, the QT interval, and the QTc interval, according to Bazett’s formula [22], were calculated for every beat within each sequence. The mean values of RR, QT, and QTc intervals were calculated in all segments of sequences, and the pre-event segments were considered the event-specific baselines. We also calculated RR, QT, and QTc intervals for a 1-min ECG segment selected during the wake stage with normal breathing for each patient to assess variations compared to a patient-specific baseline resembling normal breathing while awake. However, nine patients did not have enough continuous wake with normal breathing and thus, the analysis with patient-specific baselines involved only 120 patients.

### Statistical analysis

We utilized linear mixed-effect models to assess the effects of different parameters on mean RR, QT, and QTc intervals as dependent variables. Individual patient identifiers were included in the models as a random effect. Mixed-effect models were utilized to assess the significance of differences in RR, QT, and QTc intervals between event-specific and patient-specific baselines and other segments (segments in a sequence treated as a categorical variable with baselines as reference level), independent of confounding factors (i.e. age, sex, body mass index [BMI], and diagnosed hypertension) as fixed effects and individual patient identifiers as a random effect. In addition, mixed-effect models were incorporated to investigate the association of the presence of arousals or desaturations with the direction and magnitude of the variations in mean RR, QT, and QTc intervals. Moreover, we investigated whether parameters related to the severity of events, including the flow-limited breathing before the respiratory event (more than 50 per cent of breaths in the pre-event segment were labeled as certain flow-limited based on the procedure presented at [18]), type and duration of the respiratory event, depth of desaturation events, duration of arousals, and occurrence of respiratory events in rapid eye movement (REM) or non-rapid eye movement (NREM) sleep modulated the variations in mean RR, QT, and QTc intervals during and after the respiratory event. Sensitivity analyses were conducted to assess the effects of patients with low data contributions and the effects of different variables in the modeling. All analyses were conducted with MATLAB R2022b, with the limit for statistical significance set to *p* < .05. All *p*-values were adjusted with Bonferroni correction.

**Table 1:** Characteristics of the study population. Values are presented as number (%) or median (interquartile range) where appropriate

Clinical characteristics	
Patients (*n*, (male%))	129 (56.5)
Age (years)	45.3 (32.9, 56.4)
Body mass index (kg/m^2^)	31.7 (26.8, 38.5)
Apnea-hypopnea index (1/h)	11.3 (5.1, 26.9)
Arousal index (1/h)	23.5 (16.1, 38.4)
Oxygen desaturation index (1/h)	8.2 (1.5, 29.6)
Total sleep time (min)	342.0 (291.9, 385.1)
T90 (%)	0.2 (0.0, 5.3)
Diagnosed hypertension	29.0 (22.4)

T90 = percentage of total sleep time with oxygen saturation <90%.

## Results

### Variations in RR, QT, and QTc intervals within and after respiratory events

**Figure 2:**
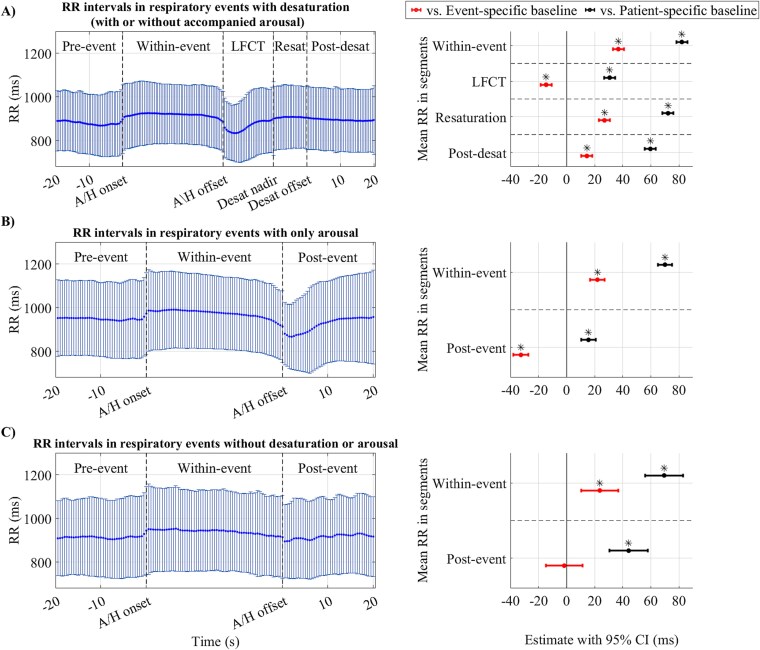
The mean and standard deviation of the RR interval series (left figures) and forest plot of linear mixed-effect model results for mean RR intervals in different segments compared to baselines (right figures) in (A) respiratory events with desaturation (with or without accompanied arousal, *n* = 2378), (B) only arousal (*n* = 1722), and (C) without desaturation or arousal (*n* = 210). Data points within segments were uniformly interpolated to provide equal-size series for visualization. Mixed-effect models were adjusted for age, sex, body mass index, and diagnosed hypertension, with patients’ identifiers as the random effect. Model estimates are presented as raw values in milliseconds. The mean RR interval in segments was considered a categorical variable with the baseline (event-specific or patient-specific) as the reference level. LFCT = lung-to-finger circulation time, Resat = resaturation, Desat = desaturation, A/H = apnea or hypopnea, CI = confidence interval. ^*^p < .05. The *p*-values are adjusted with Bonferroni correction.

The mean (standard deviation, SD) RR intervals in event-specific baselines were 920.8 (±131.1) milliseconds (ms) for events with desaturation, 969.2 (±154.7) ms for events with only arousals, and 932.5 (±164.5) ms for events without desaturation and arousals. RR intervals increased significantly in within-event segments (*p* < .05) and decreased after events (*p* < .05) compared to event-specific baselines as presented in Figure 2. However, relative RR interval variations after respiratory events without desaturation or arousal were not significantly different from the event-specific baseline (Figure 2, C). In the sequences involving desaturations, RR intervals increased again in the resaturation and post-desaturation (*p* < .05) segments compared to the event-specific baseline; however, the increase was smaller than seen in the within-event segment (Figure 2, A). Residual analysis of mixed-effect models for RR interval variations in different segments (Figure 2) is presented in Figure S3. Moreover, QT intervals showed a similar pattern of variations within different segments as RR intervals compared to event-specific baseline (mean (SD) of 407.2 (±30.8) ms for events with desaturations, 413.2 (±32.9) ms for events with only arousals, and 404.7 (±32.6) ms for events without desaturation and arousals); however, the variations were either insignificant or small (Figure S4).

**Figure 3:**
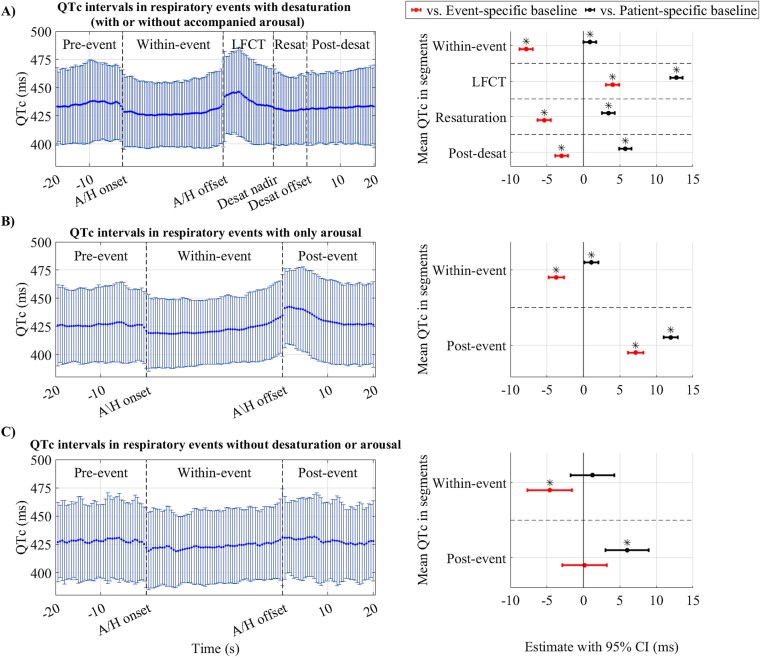
The mean and standard deviation of QTc interval series (left figures) and forest plot of linear mixed-effect model results for mean QTc intervals in different segments compared to baselines (right figures) in (A) respiratory events with desaturation (with or without accompanied arousal, *n* = 2378), (B) only arousal (*n* = 1722), and (C) without desaturation or arousal (*n* = 210). Data points within segments were uniformly interpolated to provide equal-size series for visualization. Mixed-effect models were adjusted for age, sex, body mass index, and diagnosed hypertension, with patients’ identifiers as the random effect. Model estimates are presented as raw values in milliseconds. The mean QTc interval in segments was considered a categorical variable with the baseline (event-specific or patient-specific) as the reference level. LFCT = lung-to-finger circulation time, Resat = resaturation, Desat = desaturation, A/H = apnea or hypopnea, CI = confidence interval. ^*^p < .05. The *p*-values are adjusted with Bonferroni correction.

At event-specific baselines, mean (SD) QTc values were 426.6 (±25.2) ms for events with desaturation, 422.5 (±27.3) ms for those with only arousals, and 422.5 (±29.7) ms for events without either desaturation or arousals. QTc intervals decreased significantly (*p* < .05) during respiratory events, followed by an increase in the following segment (i.e. LFCT in Figure 3A and post-event segments in Figure 3, B and C). However, the post-event QTc interval increase was negligible when the respiratory event was not followed by desaturation or arousal (Figure 3, C). In sequences with desaturations, QTc intervals showed a subsequent decrease in the resaturation and post-desaturation segments compared to the event-specific baseline (Figure 3, A). Nevertheless, this decrease was less pronounced than that for the within-event segment. Moreover, QTc intervals after respiratory events did not significantly changed when the event was not followed by desaturation and arousal (Figure 3, C). Residual analysis of mixed-effect models for QTc interval variations in different segments (Figure 3) is presented in Figure S5.

At patient-specific baselines (i.e. normal breathing while awake), the mean (SD) RR intervals were 883.6 (±128.0) ms for events with desaturations, 947.3 (±155.5) ms for events with only arousals, and 909.6 (±155.3) ms for events without desaturation and arousals. Mean (SD) of QT intervals at patient-specific baseline were 405.8 (±30.6) ms for events with desaturations, 411.7 (±32.9) ms for events with only arousals, and 403.9 (±32.6) ms for events without desaturation and arousals. Corresponding patient-specific baseline values for QTc intervals were 434.4 (±27.5) ms, 426.2 (±27.3) ms, and 426.9 (±29.7) ms, respectively. Comparison to a patient-specific baseline, while demonstrating an independent increase of RR intervals in all segments, revealed a similar pattern of between-segment variations as event-specific baseline (Figure 2, right panel figures). Moreover, QT intervals variations compared to patient-specific baseline had a similar pattern as RR intervals while, the variations were either insignificant or small (Figure S4). Considering variations from the patient-specific baseline, within-event segments had slightly higher QTc values. However, in post-event segments (LFCT and post-event), QTc values increased further and remained elevated above baseline in subsequent segments (Figure 3).

### Factors influencing the change in RR, QT, and QTc intervals

**Figure 4:**
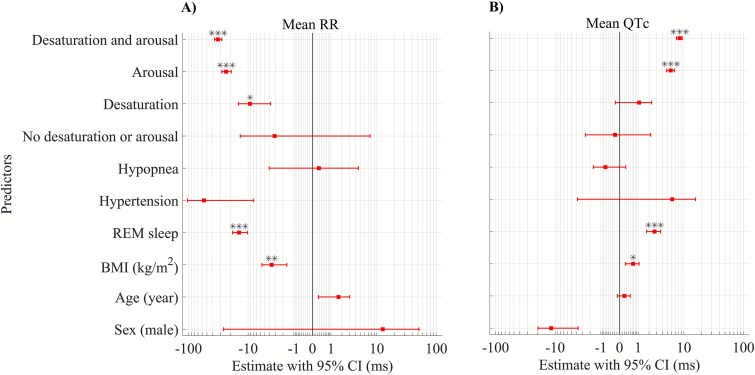
Forest plot of linear mixed-effect models for the modulatory effect of desaturations and arousals on (A) mean RR and (B) mean QTc intervals after respiratory events (LFCT segment for events with desaturations and post-event segments for events with only arousal or no desaturation and arousal). Event consequences (No desaturation and arousal [*n* = 210], desaturation [*n* = 880], arousal [*n* = 1722], or both desaturation and arousal [*n* = 1498]) are considered categorical variables with the mean pre-event values as the reference. Patients’ identifiers were included in the model as a random effect. Model estimates are presented as raw values in milliseconds. BMI = body mass index, QTc = heart rate-corrected QT interval, CI = confidence interval, ^*^p < .05, ^**^p < .01, ^***^p < .001. The *p*-values are adjusted with Bonferroni correction, and thus significance markers and non-zero-crossing 95% CIs might not coincide in some parameters.

Mixed-effect models indicate that variations in RR and QTc intervals after respiratory events are associated with desaturations and arousals after simultaneous adjustments for age, sex, BMI, respiratory event type, and hypertension, with patients’ identifiers treated as the random effect (Figure 4, residual analysis is presented in Figure S6). Our analysis revealed that when a respiratory event was not accompanied by desaturation or arousal, RR, and QTc intervals after a respiratory event did not differ significantly from those during the event-specific baseline (Figure 4). On the other hand, the highest variations in mean RR (Estimate = −32.8; 95% confidence interval (CI) = −37.3, −28.3) and mean QTc (Estimate = 8.4; 95% CI = 7.4, 9.4) intervals occurred when the respiratory event was associated with both desaturation and arousal (Figure 4). Moreover, RR intervals after respiratory events were affected by both desaturation (Estimate = −9.3; 95% CI = −14.8, −3.8) and arousal (Estimate = −23.9; 95% CI = −28.2, −19.6) while after event QTc intervals were only affected by arousal (Estimate = 5.7; 95% CI = 4.7, 6.7). However, the uncorrected QT intervals did not show significant variations in the presence or absence of desaturations and arousals (Figure S7). The sensitivity analysis also highlighted the same associations regarding the effects of desaturation and arousal on QTc and RR intervals after respiratory events (Table S1).

The type of respiratory event and the flow-limited pre-event segment were independently associated with decreased RR and increased QTc during the within-event segment (Table 2). Moreover, respiratory event duration, desaturation depth, and arousal duration were independently associated with increased QTc and decreased RR interval after respiratory events (Table 3). The associations remained the same in the sensitivity analysis (Table S4). The effects of parameters on QT intervals are presented in Tables S2 and S3. The residual analyses of models in Tables 2 and 3 are presented in Figures S8 and S9, respectively.

**Table 2:** Linear mixed-effect models for mean RR and QTc intervals within respiratory events. Values are presented as estimates (95% confidence interval)

**Predictors**	**Respiratory event without desaturation**				**Respiratory event with desaturation**			
	**RR**		**QTc**		**RR**		**QTc**	
	**Estimate (95% CI)**	***p*** **-value**	**Estimate (95% CI)**	***p*-value**	**Estimate (95% CI)**	***p*-value**	**Estimate (95% CI)**	***p*-value**
Age (year)	0.43 (−0.27, 1.13)	>.05	0.16 (0.01, 0.30)	.310	0.51 (−0.32, 1.33)	>.05	0.15 (−0.06, 0.35)	>.05
Sex (male)	13.63 (−6.12, 33.38)	>.05	**−8.50 (−12.55, −4.44)**	**<.001**	8.47 (−14.28, 31.21)	>.05	−7.82 (−13.59, −2.04)	.079
BMI (kg/m^2^)	−0.88 (−1.88, 0.11)	>.05	0.17 (−0.04, 0.37)	>.05	−0.68 (−1.84, 0.48)	>.05	0.20 (−0.09, 0.50)	>.05
REM sleep	−7.20 (−13.27, −1.14)	>.05	0.90 (−0.4, 2.2)	>.05	−0.60 (−6.54, 5.34)	>.05	0.38 (−0.90, 1.66)	>.05
Diagnosed hypertension	−22.73 (−46.91, 1.45)	>.05	1.69 (−3.24, 6.63)	>.05	−24.74 (−51.69, 2.21)	>.05	5.44 (−1.39, 12.27)	>.05
Pre-event value (ms)	**0.61 (0.58, 0.64)**	**<.001**	**0.52 (0.48, 0.55)**	**<.001**	**0.59 (0.55, 0.62)**	**<.001**	**0.36 (0.33, 0.39)**	**<.001**
Respiratory event type (hypopnea)	**−23.61 (−31.56, −15.66)**	**<.001**	**3.39 (1.69, 5.09)**	**<.001**	**−21.57 (−26.54, −16.59)**	**<.001**	**3.65 (2.58, 4.73)**	**<.001**
Respiratory event duration (s)	0.05 (−0.13, 0.23)	>.05	0.00 (−0.04, 0.04)	>.05	**0.40 (0.19, 0.60)**	**.001**	−0.04 (−0.08, 0.00)	>.05
Flow limitation	**−9.60 (−16.19, −3.01)**	**.043**	**1.68 (0.27, 3.09)**	**.019**	**−5.83 (−10.81, −0.86)**	**.021**	**1.93 (0.86, 3.01)**	**.004**

Respiratory event durations, flow-limitation status, the occurrence of events in REM sleep, and pre-event values are computed for each sequence separately; the rest of the parameters are treated patient-wise. Patients’ identifiers were included in the model as a random effect. The *p*-values are adjusted with Bonferroni correction, and thus significance and non-zero-crossing 95% CIs might not coincide in some parameters. Bold typeface font denotes *p* < .05. CI = confidence interval, QTc = heart rate-corrected QT interval, BMI = body mass index, REM = rapid eye movement sleep.

**Table 3:** Linear mixed-effect models for mean RR and QTc intervals after respiratory events. Values are presented as estimates (95% confidence interval)

**Predictors**	**Respiratory event without desaturation**				**Respiratory event with desaturation**			
	**RR**		**QTc**		**RR**		**QTc**	
	**Estimate (95% CI)**	***p*-value**	**Estimate (95% CI)**	***p*-value**	**Estimate (95% CI)**	***p*-value**	**Estimate (95% CI)**	***p*-value**
Age (year)	1.02 (0.26, 1.77)	>.05	0.01 (−0.13, 0.16)	>.05	0.78 (−0.07, 1.62)	>.05	0.15 (−0.07, 0.38)	>.05
Sex (male)	4.45 (−16.92, 25.82)	>.05	−4.76 (−8.96, −0.55)	>.05	6.45 (−16.86, 29.77)	>.05	−6.40 (−12.56, −0.25)	>.05
BMI (kg/m^2^)	−1.34 (−2.42, −0.26)	>.05	0.24 (0.03, 0.45)	>.05	−1.15 (−2.34, 0.04)	>.05	0.31 (0.00, 0.63)	>.05
REM sleep	**−16.17 (−25.03, −7.31)**	**.003**	2.33 (0.40, 4.25)	>.05	**−17.16 (−24.2, −10.12)**	**<.001**	**3.72 (1.99, 5.45)**	**<.001**
Diagnosed hypertension	−24.88 (−51.03, 1.27)	>.05	1.60 (−3.48, 6.68)	>.05	−19.93 (−47.51, 7.65)	>.05	3.50 (−3.76, 10.75)	>.05
Pre-event value (ms)	**0.53 (0.48, 0.57)**	**<.001**	**0.56 (0.52, 0.61)**	**<.001**	**0.50 (0.46, 0.54)**	**<.001**	**0.37 (0.33, 0.41)**	**<.001**
Respiratory event type (hypopnea)	−4.29 (−15.99, 7.41)	>.05	0.94 (−1.60, 3.49)	>.05	**−11.98 (−18.19, −5.78)**	**.002**	**2.96 (1.43, 4.48)**	**.001**
Respiratory event duration (s)	**−0.62 (−0.88, −0.36)**	**<.001**	**0.12 (0.07, 0.18)**	**<.001**	**−0.46 (−0.71, −0.21)**	**.002**	**0.18 (0.12, 0.24)**	**<.001**
Desaturation depth (%)	-	-	-	-	**−0.09 (−0.16, −0.02)**	**.048**	**0.56 (0.41, 0.72)**	**<.001**
Arousal duration (s)	**−5.71 (−6.31, −5.12)**	**<.001**	**1.25 (1.12, 1.38)**	**<.001**	**−3.52 (−3.94, −3.09)**	**<.001**	**0.84 (0.74, 0.94)**	**<.001**

Respiratory event durations, the occurrence of events in REM sleep, pre-event values, desaturation depth, and arousal duration are computed for each sequence separately; the rest of the parameters are treated patient-wise. Patients’ identifiers were included in the model as a random effect. The *p*-values are adjusted with Bonferroni correction, and thus significance and non-zero-crossing 95% CIs might not coincide in some parameters. Bold typeface font denotes *p* < .05. CI = confidence interval, QTc = heart rate-corrected QT interval, BMI = body mass index, REM = rapid eye movement sleep.

## Discussion

This study examined the temporal dynamics of cardiac response to respiratory events and focused on the potential modulators of this response. Our data indicate that the relative changes in post-event cardiac electrical activity are associated with the presence of desaturations and arousals. We also observed that more severe desaturations and longer arousals increases the magnitude of change in cardiac electrical activity after respiratory events. Our results provide new insights regarding the modulators of post-respiratory event cardiac electrical activity, thereby contributing to understanding the possible mechanisms explaining the increased risk of arrhythmogenesis in OSA patients. Moreover, we identified REM sleep as a more vulnerable phase for cardiac alterations that NREM sleep when related to sleep apnea events.

### Physiological insights

Alterations in the ECG waveform due to respiratory events can originate from several physiological reflex mechanisms. Airway obstruction and hypoxia related to respiratory events activate the diving reflex, increasing cardiac parasympathetic and peripheral vascular sympathetic activity [23], and subsequent parasympathetic tone withdrawal at the termination of the respiratory events can lead to immediate cardiac electrical response [6]. Moreover, increased intrathoracic pressure swings during respiratory events has been related to sympathetic activity via mechano-receptor feedback within the heart [7] and respiratory event-induced hypoxemia and hypercapnia activate peripheral and central chemoreceptors, leading to sympathetic activation [24]. Furthermore, respiratory event-related arousals may elicit acute transient sympathetic activations [25], and systolic and diastolic blood pressure surges following arousals may lead to baroreceptor stimulation [26], both contributing to cardiac electrical activity alterations. We consistently observed increased RR and decreased QTc intervals during respiratory events compared to the event-specific baselines. Yet, RR and QTc intervals after respiratory events were not significantly different from event-specific baselines when desaturations or arousals were absent. This may suggest that when respiratory events are not followed by desaturations or arousals, the resulting intrathoracic pressure changes are insufficient to overtly impact our cardiac electrophysiologic measures. However, desaturation and arousal decreased RR and increased QTc intervals after respiratory events compared to the event-specific baselines (Figures 2 and 3). Our observations align with previous studies on general RR interval alterations due to respiratory events [10–12]. Respiratory event termination with arousal was shown to be accompanied by hyperventilation [27]. Post-event heart rate acceleration in the absence of cortical arousals has previously been attributed to the subcortical reflex response [27, 28]. However, in the current study, these responses were only present in association with a post-respiratory event desaturation and may suggest a possible link between desaturation events and subcortical arousal.

Although we observed the largest relative post-event cardiac response in the presence of both desaturation and arousal, a previous study showed that cardiac response to auditory arousals did not differ between hypercapnic hypoxia and normoxia conditions [29]. This discrepancy can originate from different degrees of hypoxia when compared to controlled ventilation conditions, as our analysis indicated the independent effect of desaturation depth on cardiac response. In addition, observed QTc intervals during respiratory events contradict an earlier study that presented increased QTc intervals during respiratory events compared to pre-event intervals [30]. However, our observations are based on a larger pool of data and provide evidence of distinct respiratory event-related cardiac responses independent of confounding factors and intersubject variability. Our findings highlight the major role of heart rhythm neural controls compared to direct electrophysiological changes after respiratory events, as evident by significant changes in RR intervals and small QT interval alterations. This potentially suggests elevated sympathetic activity when respiratory events trigger both desaturation and arousal.

Dynamics of cardiac electrical activity during and after respiratory events were observed to be affected by confounding factors. We observed a consistent independent modulatory effect of pre-respiratory event flow-limited breathing on RR and QTc intervals within respiratory events (Table 2). Although previous studies indicated that flow-limited breathing can be accompanied by an independent cardiac response [31], we focused on the possible accumulated effects of flow-limited breathing on cardiac variations during the respiratory events. Flow-limited breathing can initiate altered intrathoracic pressure swings before the initiation of respiratory events, thus leading to more pronounced cardiac activity within the events, as we observed in our analysis. Moreover, respiratory events in REM sleep were associated with significantly decreased RR interval and increased QTc interval after respiratory events compared to NREM sleep (Table 3). While sleep stage-specific differences in RR and QTc intervals have been previously observed [32]; there is mixed evidence about the impact of sleep state on post-event electrical activity, with both REM sleep-related modulation [5] and insignificant effects of sleep stages [12] being reported. REM sleep is associated with increased sympathetic activity, whereas NREM sleep is associated with parasympathetic activity, which progressively increases with deeper NREM stages [25]. Although our results presented significant effects of REM sleep compared to NREM sleep in respiratory event-related cardiac responses, inclusion of all sleep stages (N1, N2, N3, and REM sleep) destabilized statistical modeling, potentially due to unequal/insufficient data size for all sleep stages. Therefore, further studies are required to better understand the effect of all sleep stages on respiratory event-related cardiac responses. Furthermore, as a common OSA comorbidity, hypertension was not observed to have a significant effect on cardiac electrical activity during and after respiratory events (Tables 2 and 3). This may be due to modeling hypertension as a dichotomous variable without accounting for its severity, varying degrees of control of hypertension, the influences of antihypertensive medications, and the low prevalence of the disease in the study population.

### Clinical implications

We found that the presence of desaturation and arousal are independently associated with cardiac electrical function, with the greatest relative changes in RR and QTc intervals occurring when both factors are present; and conversely, that differences between post-event cardiac electrical activity and event-specific baselines in the absence of related desaturation and arousal were not statistically significant (Figure 4). This is consistent with prior reports showing the possible modulatory role of arousal and deep desaturations on cardiac autonomic function [12], and that desaturation and arousal have independent effects on ventricular repolarization in OSA patients [13, 15, 33]. These data suggest that measures of desaturation and arousal severity may correlate better with the risk of nocturnal cardiovascular events than the conventional OSA severity metrics. This is consistent with recent studies showing that the overall hypoxemic load and arousal burden better predict cardiovascular mortality than the AHI [34–37]. However, in another study, incorporation of arousal associated respiratory events did not predict incident cardiovascular disease better than a desaturation associated respiratory event [38].

Previous studies have demonstrated that longer respiratory events are associated with a greater decrease in RR intervals after respiratory events compared to shorter events [11, 12], that deeper desaturations are associated with increased ventricular repolarization lability [13], and that increased arousal intensity and arousal duration are associated with increased cardiac activity [39, 40]. Consistent with previous literature, our analysis shows that the duration of respiratory events, deeper desaturation, and longer arousal are independently associated with the magnitude of RR interval decrease and QTc interval increase in post-event segments compared to event-specific baselines (Table 3). This relationship may explain, at least in part, the significantly elevated risk of arrhythmia after respiratory events [4]. Our observation also suggests a possible mechanism behind the elevated risk of arrhythmogenesis after respiratory events through observed fast-paced variations in cardiac electrical activity. The occurrence of several respiratory events in close succession can potentially influence the QT-RR adaptation mechanism. The QT-RR adaptation, i.e. the dynamic adjustment of the QT intervals in response to changes in the heart rate [41], could be influenced by the rapid changes due to the respiratory event-related cardiac electrical activity. However, a detailed analysis of QT-RR adaptations during and after respiratory events is warranted. Notably, our results are based on a population with moderate sleep apnea, low T90, no medications, and no comorbidities except diagnosed hypertension. This further highlights the importance of our results: even patients with milder OSA burden are prone to acute cardiac electrical activity alterations if they experience severe respiratory events during sleep.

### Limitations

Our exclusion criteria led to omitting a large pool of patients, affecting the generalizability of the present findings. However, excluding patients with comorbidities and medications known to affect cardiac electrophysiology was done to minimize the effects of confounding factors and pre-existing cardiovascular alterations. We also excluded events occurring in close proximity to each other and those where desaturation ended during respiratory events to better understand the modulatory mechanisms of cardiac electrical activity during and after respiratory events. Thus, these results may not generalize to periods of consecutive respiratory events or individuals who primarily experience consecutive respiratory events in close proximity. Moreover, the number of data sequences available for analysis was heavily affected by our strict exclusion criteria to minimize potential misinterpretation of the observations; therefore, no power analysis was performed. Our focus on cardiac response to isolated events and modulators of its response are necessary to fully understand the temporal dynamics of alterations unaffected by responses to adjacent events. In addition, we did not consider the effects of transient blood pressure alteration within the respiratory event cycles due to the unavailability of blood pressure measurement within the standard PSG setup. Dose–response effect of blood pressure alteration on arousal intensity [42] and worsening hypoxemia [43] might further increase our understanding regarding respiratory events-related alterations, yet we believe the lack of this information does not jeopardize our results. Additionally, our heartbeat delineation algorithm is fully automated, which can be prone to artifacts and misdetections. We addressed this by inspecting signal quality, identifying T-wave abnormalities, and excluding noisy segments to reduce the possible inclusion of artifacts in the analysis. Furthermore, our dataset lacks a clinical endpoint, and our results cannot directly be related to cardiovascular outcomes. Despite this, our results present compelling evidence of possible modulators of post-event cardiac electrical activity. Our observations provide evidence that due to transient dynamics of respiratory events, patients with high hypoxic load and fragmented sleep might have a higher tendency to QTc prolongation.

## Conclusion

Based on the present results, respiratory events in OSA patients are associated with significant cardiac variations if they lead to desaturations and arousals. Moreover, the duration of respiratory events, depth of desaturations, and duration of arousals were independently associated with greater cardiac response. These findings indicate that not all respiratory events have a similar effect on the heart, and the magnitude of the impact is related to the presence and severity of related desaturations and arousals.

## Supplementary Material

Supplementary_file_zsaf382

## Data Availability

Data cannot be shared publicly because of potentially identifying or sensitive patient information. These ethical restrictions are imposed by the Institutional Human Research Ethics Committee of the Princess Alexandra Hospital. Data are available from the Institutional Human Research Ethics Committee of the Princess Alexandra Hospital (contact via MSHEthics@health.qld.gov.au) for researchers who meet the criteria for access to confidential data. Researchers can contact the IHREC of PA Hospital and the project steering committee will review the requests.
