# Supplementary material for: Hypoxemia and arousals modulate cardiac responses to respiratory events in obstructive sleep apnea
Source: Sleep. 2025 Dec 1;49(3):zsaf382. doi: 10.1093/sleep/zsaf382 (PMC13017676; doi:10.1093/sleep/zsaf382)
Supplement: Supplementary_file_zsaf382 [file supplementary_file_zsaf382.docx]

**Hypoxemia and arousals modulate cardiac responses to respiratory events in obstructive sleep apnea**

Serajeddin Ebrahimian^1,2^, Saara Sillanmäki^2,3^, Marika Rissanen^1,2^, Eric Staykov^4^, Antti Kulkas^1,5^, Juha Töyräs^1,4,6^, Raquel Bailón^7,8^, Ludger Grote^9^, Maria R Bonsignore^10^, Mathias Baumert^11^, Virend K Somers^12^, Philip Terrill^4^, Timo Leppänen^1,2,4^, Samu Kainulainen^1,2^

^1^ Department of Technical Physics, University of Eastern Finland, Kuopio, Finland

^2^ Diagnostic Imaging Center, Kuopio University Hospital, Kuopio, Finland

^3^ Institute of Clinical Medicine, University of Eastern Finland, Kuopio, Finland

^4^ School of Electrical Engineering and Computer Science, The University of Queensland, Brisbane, Australia

^5^ Department of Clinical Neurophysiology, Seinäjoki Central Hospital, Seinäjoki, Finland

^6^ Science Service Center, Kuopio University Hospital, Kuopio, Finland

^7^ Biomedical Signal Interpretation and Computational Simulation (BSICoS) Group, Aragón Institute of Engineering Research (I3A), IIS Aragón, University of Zaragoza, Zaragoza, Spain.

^8^ Centro de Investigación Biomédica en Red en Bioingeniería, Biomateriales y Nanomedicina (CIBER-BBN), Madrid, Spain

^9^ Centre for Sleep and Vigilance Disorders, Sahlgrenska Academy, Gothenburg University, Gothenburg, Sweden

^10^ PROMISE Department, University of Palermo and IFT-CNR, Palermo, Italy

^11^ School of Electrical and Mechanical Engineering, The University of Adelaide, Adelaide, Australia

^12^Department of Cardiovascular Medicine, Mayo Clinic, Rochester MN, USA

**Corresponding author:**

Serajeddin Ebrahimian, M.Sc.

Department of Technical Physics, University of Eastern Finland

P.O. Box 1627 (Canthia), 70211 Kuopio

serajeddin.ebrahimian@uef.fi

Tel: +358458092688


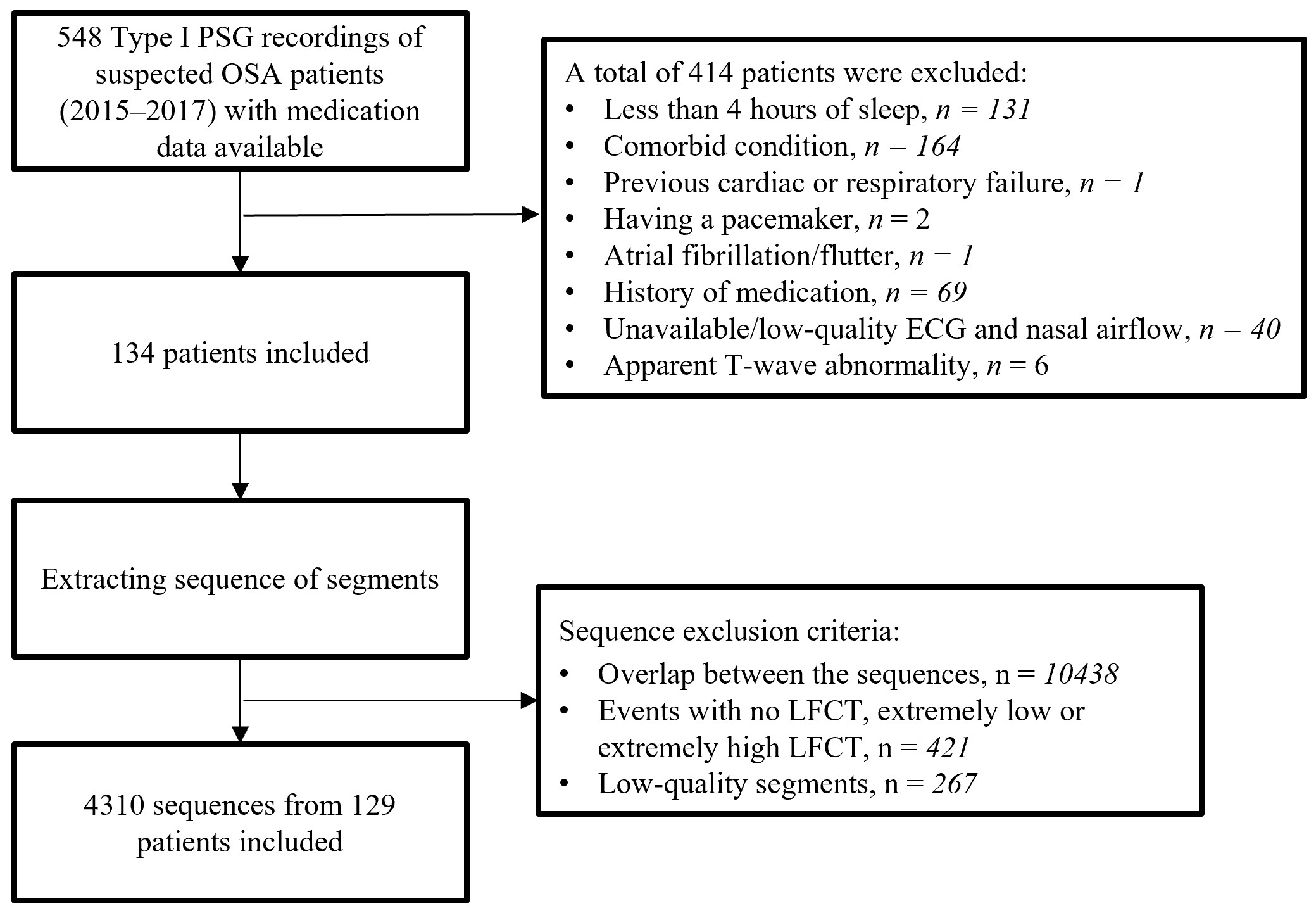


**Figure S1**. Flowchart of exclusion criteria for patients and individual events. PSG = polysomnography, OSA = obstructive sleep apnea, ECG = electrocardiogram, LFCT = lung-to-finger circulation time


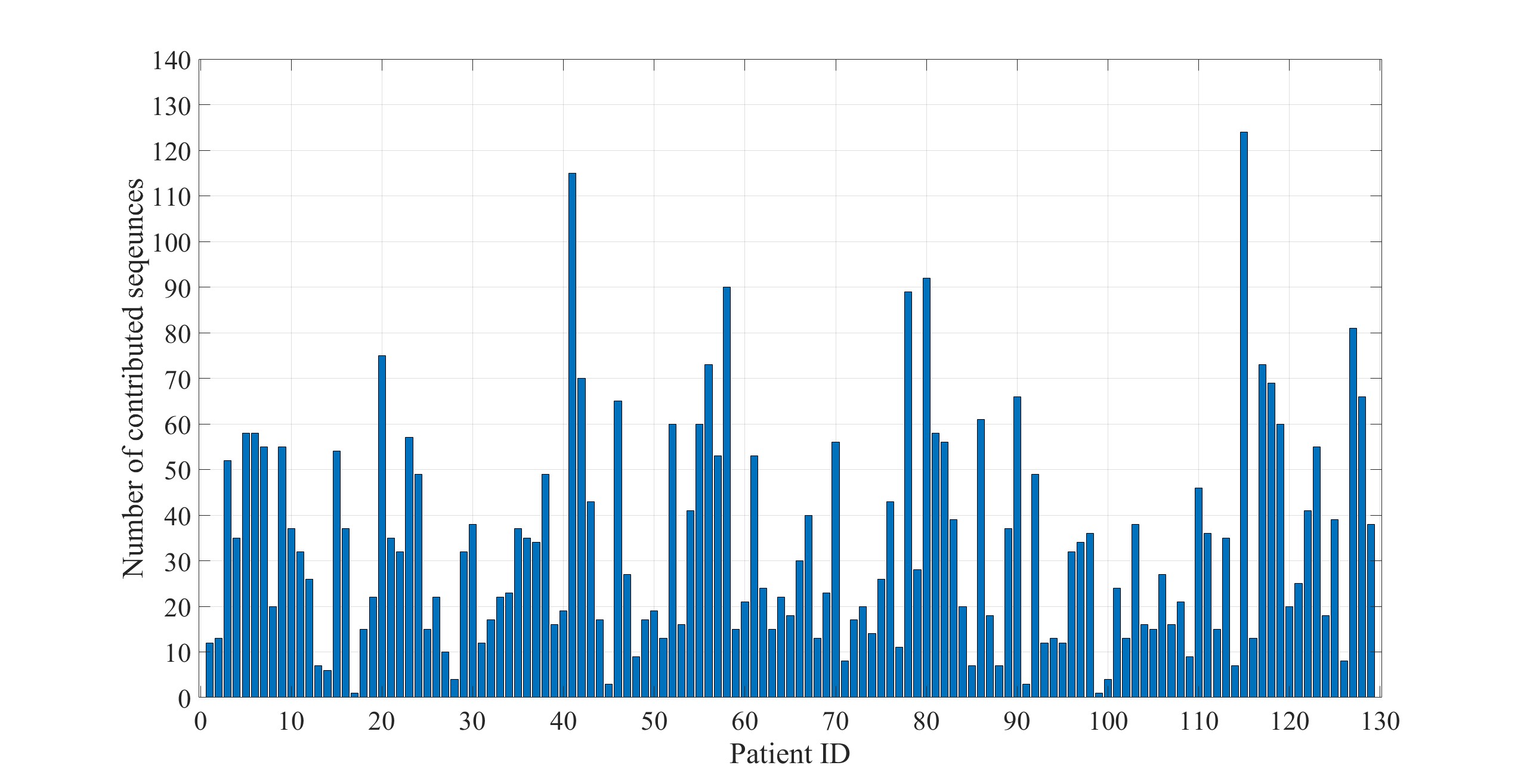


**Figure S2.** Number of sequences in the analysis from each patient. Each histogram bar represents an individual study subject (*N* = 129). The y-axis shows the number of sequences included in the analysis for each patient. ID = study subject identifier.


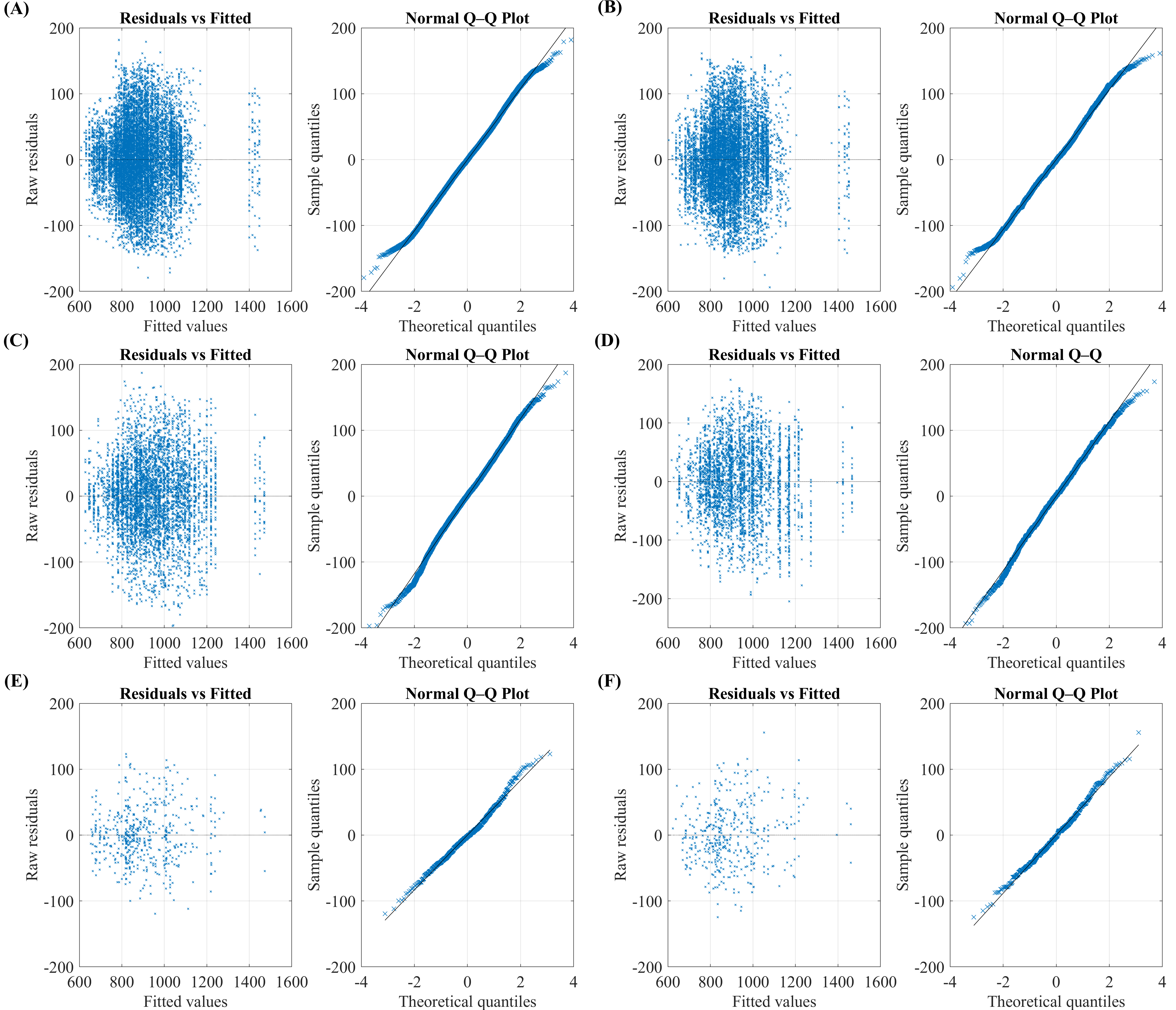


**Figure S3**. Residuals of modeling RR interval variations within segments compared to (A, C, E) event-specific and (B, D, F) patient-specific baselines for (A, B) respiratory events with desaturations, (C, D) respiratory events with only arousals, and (E, F) for respiratory events without desaturations and arousals. For all models, residuals do not indicate problematic patterns in Residual vs. Fitted plots and Q-Q plots showing residuals are approximately normal.

**
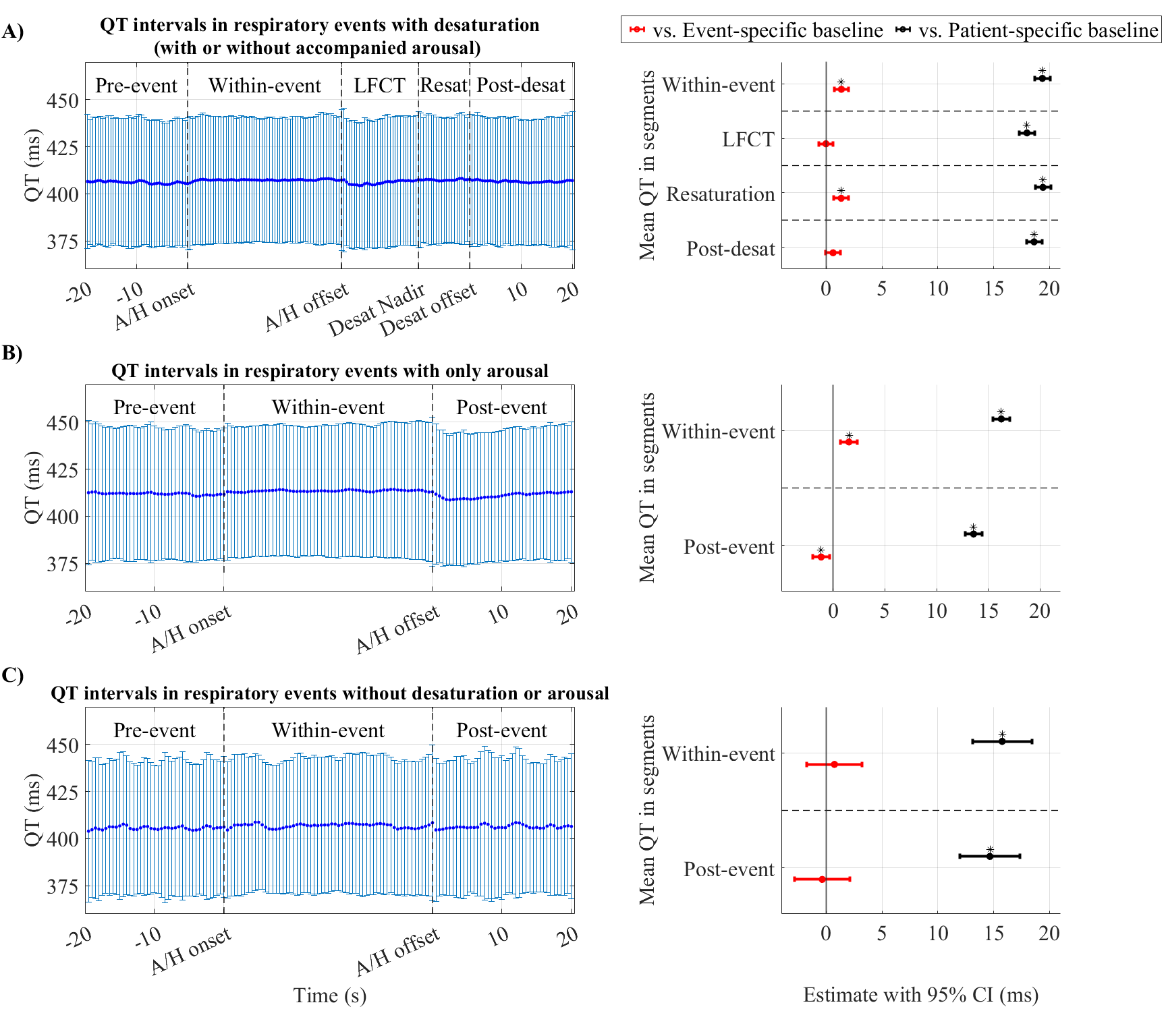
 Figure S4.** The mean and standard deviation of the QT interval series (left figures) and forest plot of linear mixed effect model results for mean QT intervals in different segments compared to baselines (right figures) in (A) respiratory events with desaturation (with or without accompanied arousal, n = 2378), (B) only arousal (n = 1722), and (C) without desaturation or arousal (n = 210). Data points within segments were uniformly interpolated to provide equal-size series for visualization. Mixed effect models were adjusted for age, sex, body mass index, and diagnosed hypertension, with patients’ identifiers as the random effect. Model estimates are presented as raw values in milliseconds. The mean QT interval in segments was considered a categorical variable with the baseline (event-specific or patient-specific) as the reference level. LFCT = lung-to-finger circulation time, Resat = resaturation, Desat = desaturation, A/H = apnea or hypopnea, CI = confidence interval. * = *p* < 0.05. The *p*-values are adjusted with Bonferroni correction.


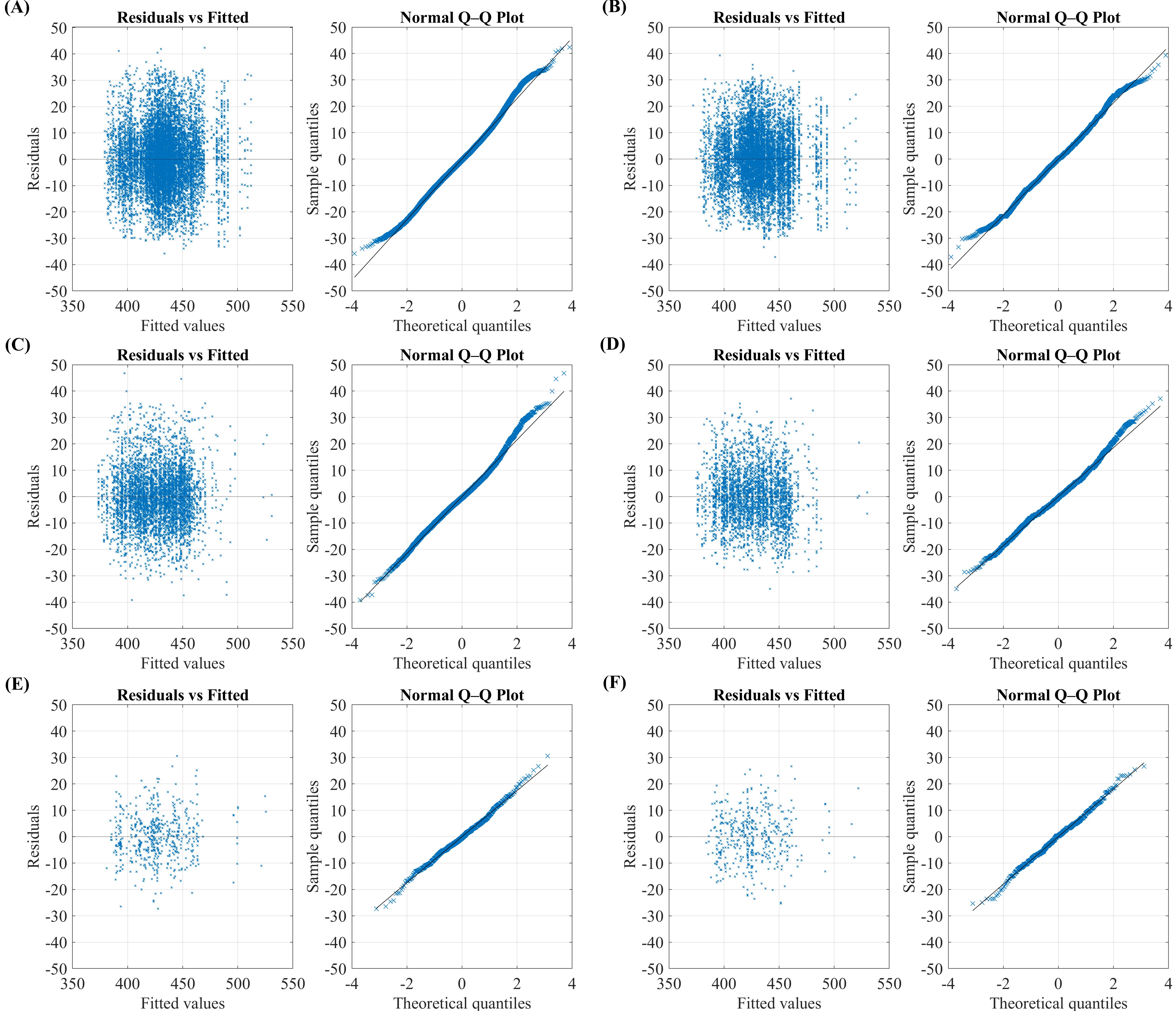
**Figure S5**. Residuals of modeling QTc interval variations within segments compared to (A, C, E) event-specific and (B, D, F) patient-specific baselines for (A, B) respiratory events with desaturations, (C, D) respiratory events with only arousals, and (E, F) for respiratory events without desaturations and arousals. For all models, residuals do not indicate problematic patterns in Residual vs. Fitted plots and Q-Q plots showing residuals are approximately normal.


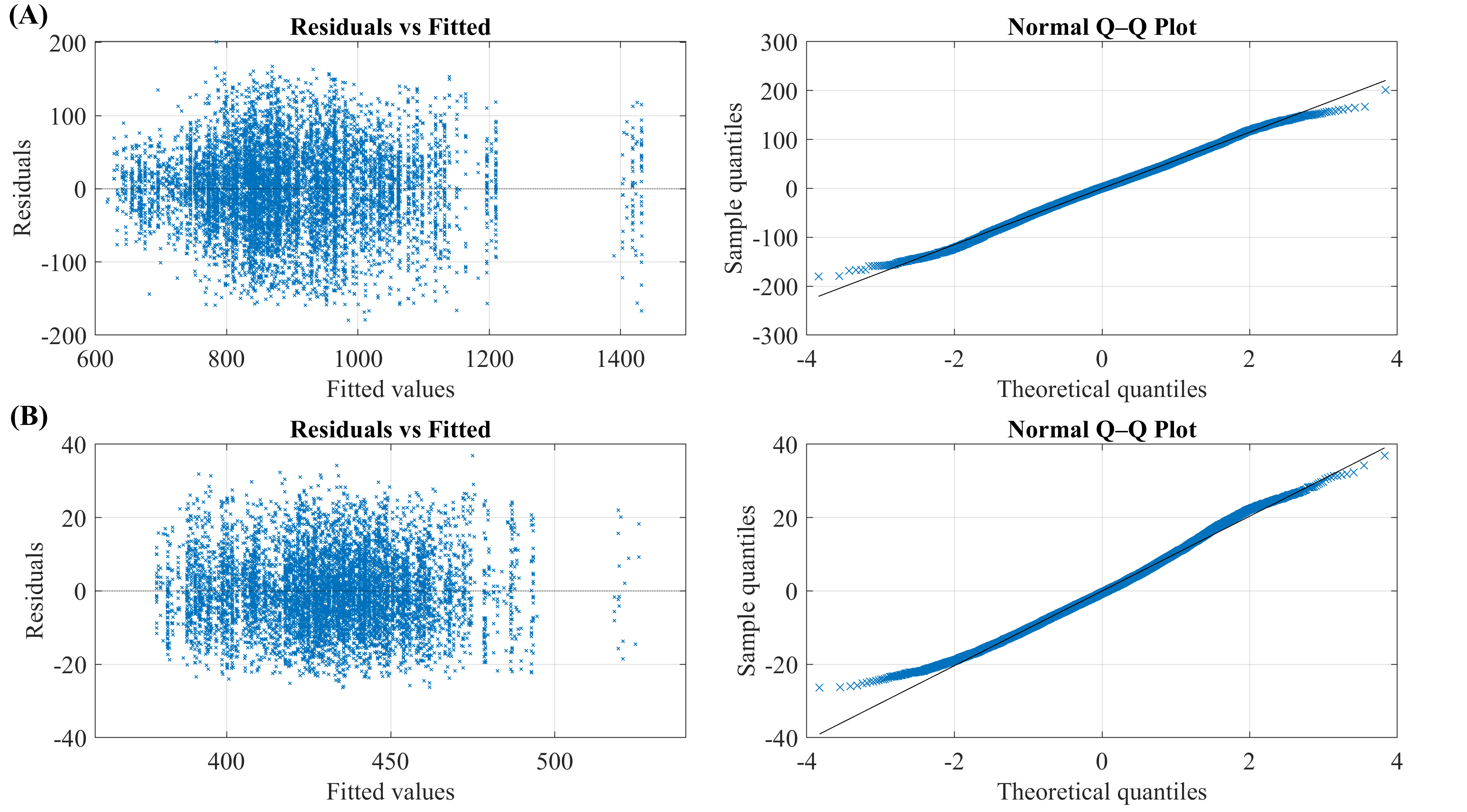


**Figure S6**. Residual analysis of mixed effect models for the modulatory effect of desaturations and arousals on (A) mean RR and (B) mean QTc intervals after respiratory events (related to Figure 4 of the manuscript). For both models, residuals do not indicate problematic patterns in Residual vs. Fitted plots and Q-Q plots showing residuals are approximately normal.

**Figure S7**. Forest plot of linear mixed effect models for the modulatory effect of desaturations and arousals on mean QT intervals after respiratory events (LFCT segment for events with desaturations and post-event segments for events with only arousal or no desaturation and arousal). Event consequences (No desaturation and arousal [*n* = 210], desaturation [*n* = 880], arousal [*n* = 1722], or both desaturation and arousal [*n* = 1498]) are considered categorical variables with the mean pre-event values as the reference. Patients’ identifiers were included in the model as a random effect. Model estimates are presented as raw values in milliseconds. BMI = body mass index, CI = confidence interval, * = *p* < 0.05. The *p*-values are adjusted with Bonferroni correction.


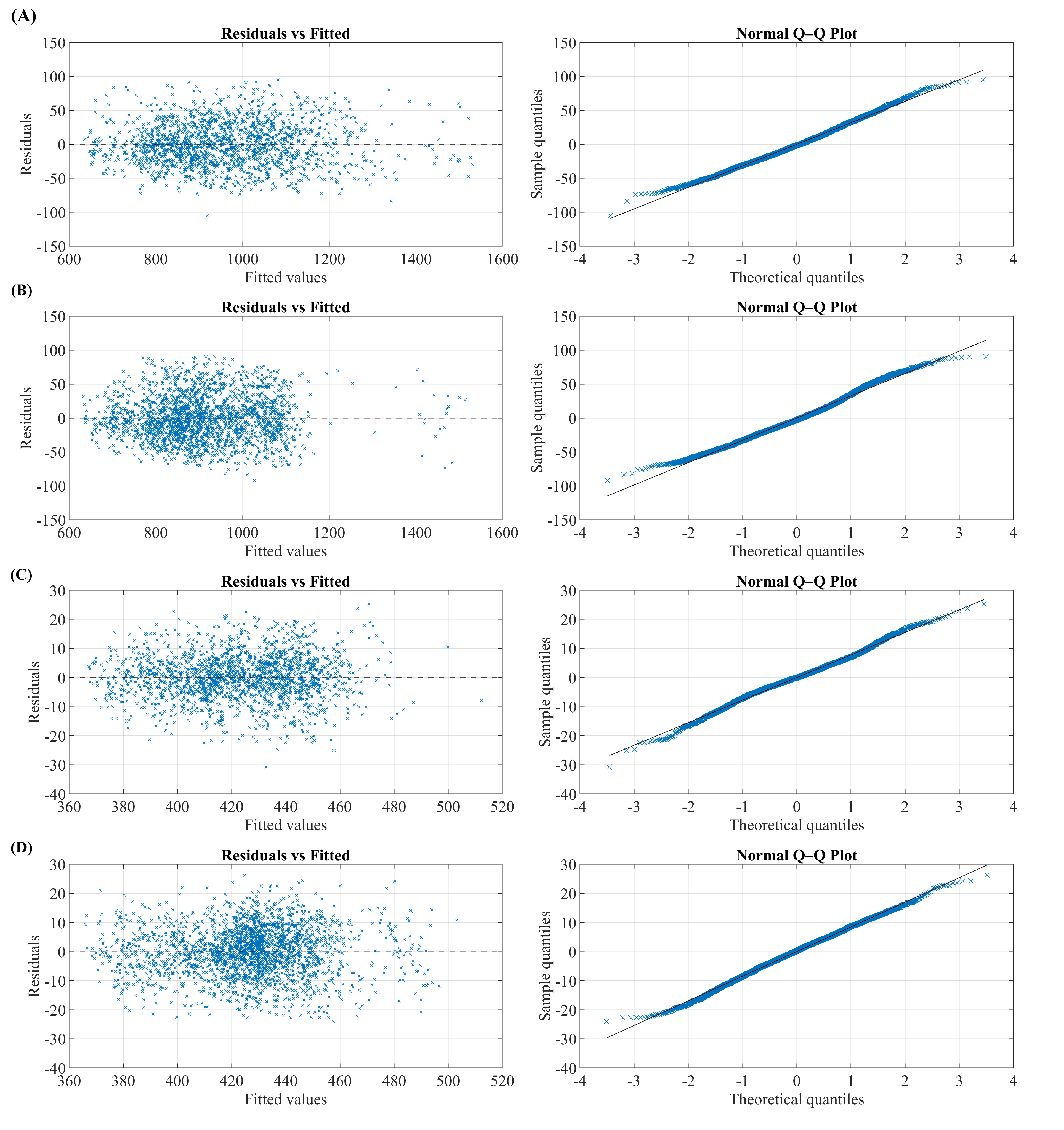


**Figure S8.** Residual analysis of mixed effect models for mean RR and QTc intervals within respiratory events (related to Table 2 of the manuscripts). Residuals for (A) RR intervals in respiratory events without desaturation and (B) with desaturation. Residuals for (C) QTc intervals in respiratory events without desaturation and (D) with desaturation. For all models, residuals do not indicate problematic patterns in Residual vs. Fitted plots and Q-Q plots showing residuals are approximately normal.


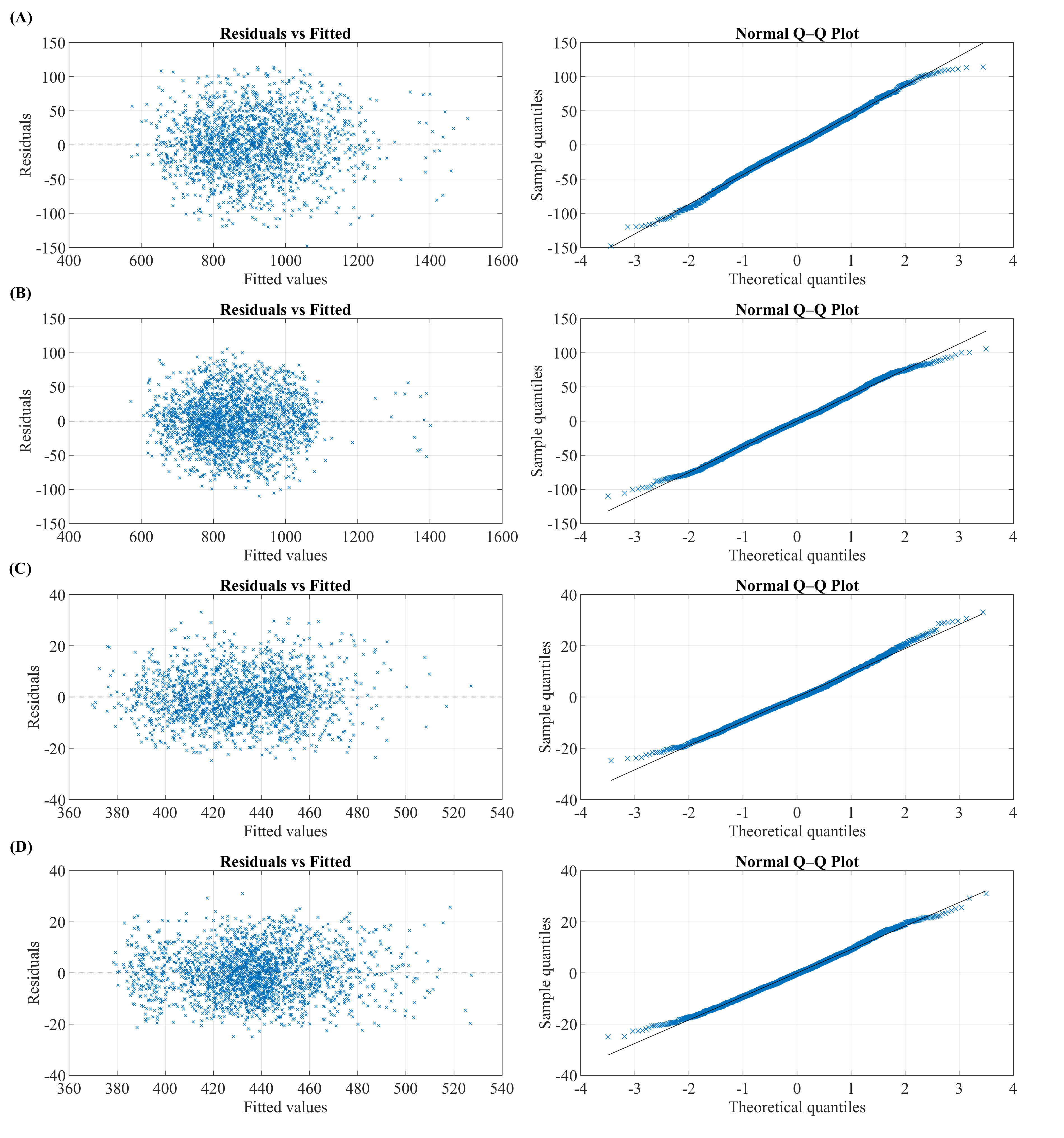


**Figure S9.** Residual analysis of mixed effect models for mean RR and QTc intervals after respiratory events (related to Table 3 of the manuscripts). Residuals of modeling (A) RR intervals in respiratory events without desaturation and (B) with desaturation. Residuals of modeling (C) QTc intervals in respiratory events without desaturation and (D) with desaturation. For all models, residuals do not indicate problematic patterns in Residual vs. Fitted plots and Q-Q plots showing residuals are approximately normal.

**Table S1**. Sensitivity analysis of the effects of desaturations and arousals on post-respiratory event RR and QTc values after exclusion of patients with low data contribution and removal of confounders from the model. Values are presented as estimates (95% confidence interval)

| Predictor | RR | QTc |
| --- | --- | --- |
| Exclusion of patients with fewer than 5 sequences (n=6) | | |
| Desaturation and arousal | **-32.76 (-37.26, -28.26)** | **8.4 (7.4, 9.4)** |
| Arousal | **-23.79 (-28.11, -19.47)** | **5.7 (4.73, 6.67)** |
| Desaturation | **-9.25 (-14.78, -3.72)** | 1.1 (-0.14, 2.34) |
| No desaturation and arousal | -3.06 (-13.71, 7.59) | -0.18 (-2.58, 2.22) |
| Exclusion of patients with fewer than 10 sequences (n=15) | | |
| Desaturation and arousal | **-32.53 (-37.03, -28.03)** | **8.38 (7.37, 9.39)** |
| Arousal | **-23.55 (-27.95, -19.15)** | **5.7 (4.72, 6.68)** |
| Desaturation | **-9.1 (-14.6, -3.6)** | 1.06 (-0.18, 2.3) |
| No desaturation and arousal | -2.8 (-13.4, 7.8) | -0.23 (-2.62, 2.16) |
| Removing sex variable | | |
| Desaturation and arousal | **-32.53 (-37.04, -28.02)** | **8.37 (7.36, 9.38)** |
| Arousal | **-23.55 (-27.92, -19.18)** | **5.7 (4.72, 6.68)** |
| Desaturation | **-9.08 (-14.63, -3.53)** | 1.06 (-0.18, 2.3) |
| No desaturation and arousal | -2.82 (-13.47, 7.83) | -0.22 (-2.61, 2.17) |
| Removing BMI variable | | |
| Desaturation and arousal | **-32.53 (-37.93, -27.13)** | **8.37 (7.36, 9.38)** |
| Arousal | **-23.43 (-27.78, -19.08)** | **5.67 (4.69, 6.65)** |
| Desaturation | **-8.77 (-14.29, -3.25)** | 0.98 (-0.26, 2.22) |
| No desaturation and arousal | 2.78 (1.12, 4.44) | -0.24 (-2.64, 2.16) |
| Removing event type variable | | |
| Desaturation and arousal | **-32.54 (-37.03, -28.05)** | **8.45 (7.45, 9.45)** |
| Arousal | **-23.54 (-27.9, -19.18)** | **5.64 (4.67, 6.61)** |
| Desaturation | **-9.07 (-14.62, -3.52)** | 1.02 (-0.23, 2.27) |
| No desaturation and arousal | -2.84 (-4.49, -1.19) | -0.17 (-2.55, 2.21) |
| Removing hypertension variable | | |
| Desaturation and arousal | **-32.53 (-37.04, -28.02)** | **8.38 (7.37, 9.39)** |
| Arousal | **-23.65 (-28.02, -19.28)** | **5.69 (4.71, 6.67)** |
| Desaturation | **-9.08 (-14.63, -3.53)** | 1.06 (-0.19, 2.31) |
| No desaturation and arousal | -2.83 (-5.48, -0.18) | -0.23 (-2.62, 2.16) |

Mixed effect models were adjusted for age, sex, body mass index, sleep-stage (NREM vs. REM), and diagnosed hypertension, with patients as the random effect. Model estimates are presented as raw values in milliseconds. The *p*-values are adjusted with Bonferroni correction. Bold typeface font denotes *p* < 0.05. BMI = body mass index, QTc = heart rate corrected QT interval.

**Table S2**. Linear mixed effect models for mean QT intervals within respiratory events. Values are presented as estimates (95% confidence interval).

| Predictors | Respiratory event without desaturation | | Respiratory event with desaturation | |
| --- | --- | --- | --- | --- |
|  | **QT** | ***p*-values** | **QT** | ***p*-value** |
| **Age (year)** | 0.01 (-0.02, 0.04) | >0.05 | 0.05 (-0.02, 0.13) | >0.05 |
| **Sex (male)** | -0.85 (-1.72, 0.01) | >0.05 | -1.39 (-3.35, 0.56) | >0.05 |
| **BMI (kg/m^2^)** | -0.01 (-0.06, 0.03) | >0.05 | -0.05 (-0.15, 0.05) | >0.05 |
| **REM sleep** | -0.49 (-1.2, 0.22) | >0.05 | 0.01 (-0.74, 0.76) | >0.05 |
| **Diagnosed hypertension** | -0.99 (-2.03, 0.05) | >0.05 | -0.44 (-2.72, 1.84) | >0.05 |
| **Pre-event value (ms)** | **0.97 (0.96, 0.98)** | **<0.001** | **0.85 (0.83, 0.87)** | **<0.001** |
| **Respiratory event type**  **(hypopnea)** | **-1.41 (-2.35, -0.48)** | **0.030** | **-0.90 (-1.53, -0.27)** | **0.05** |
| **Respiratory event duration (s)** | 0.00 (-0.02, 0.02) | >0.05 | 0.03 (0.00, 0.06) | >0.05 |
| **Flow limitation** | -0.80 (-1.56, -0.05) | >0.05 | 0.11 (-0.52, 0.75) | >0.05 |

Respiratory event durations, flow-limitation status, the occurrence of events in REM sleep, and pre-event values are computed for each sequence separately; the rest of the parameters are treated patient-wise. The *p*-values are adjusted with Bonferroni correction, and thus significance and non-zero-crossing 95% CIs might not coincide in some parameters. Bold typeface font denotes *p* < 0.05. BMI = body mass index, REM = rapid eye movement sleep.

**Table S3**. Linear mixed effect models for mean QT intervals after respiratory events. Values are presented as estimates (95% confidence interval).

| Predictors | Respiratory event without desaturation | | Respiratory event with desaturation | |
| --- | --- | --- | --- | --- |
|  | **QT** | ***p*-values** | **QT** | ***p*-values** |
| **Age (year)** | **0.09 (0.04, 0.13)** | **0.003** | **0.11 (0.04, 0.18)** | **0.022** |
| **Sex (male)** | -0.98 (-2.29, 0.34) | >0.05 | 0.05 (-1.89, 1.98) | >0.05 |
| **BMI (kg/m^2^)** | 0.01 (-0.06, 0.07) | >0.05 | 0.01 (-0.08, 0.11) | >0.05 |
| **REM sleep** | **-1.31 (-2.13, -0.49)** | **0.017** | -0.08 (-1.01, 0.84) | >0.05 |
| **Diagnosed hypertension** | -0.65 (-2.24, 0.94) | >0.05 | -0.20 (-2.44, 2.03) | >0.05 |
| **Pre-event value (ms)** | **0.90 (0.89, 0.92)** | **<0.001** | **0.86 (0.83, 0.88)** | **<0.001** |
| **Respiratory event type (hypopnea)** | 0.02 (-1.07, 1.10) | >0.05 | -0.35 (-1.17, 0.47) | >0.05 |
| **Respiratory event duration (s)** | -0.02 (-0.04, 0.01) | >0.05 | 0.04 (0.00, 0.07) | >0.05 |
| **Desaturation depth (%)** | - | **-** | **0.23 (0.15, 0.32)** | **<0.001** |
| **Arousal duration (s)** | **-0.21 (-0.26, -0.15)** | **<0.001** | **-0.06 (-0.12, -0.01)** | **<0.001** |

Respiratory event durations, the occurrence of events in REM sleep, pre-event values, desaturation depth, and arousal duration are computed for each sequence separately; the rest of the parameters are treated patient-wise. The *p*-values are adjusted with Bonferroni correction, and thus significance and non-zero-crossing 95% CIs might not coincide in some parameters. Bold typeface font denotes *p* < 0.05. BMI = body mass index, REM = rapid eye movement sleep.

**Table S4**. Sensitivity analysis of post-event mean RR and QTc intervals after exclusion of patients with low data contribution and removal of confounders from the model. Values are presented as estimates (95% confidence interval).

| Predictors | Respiratory event without desaturation | | Respiratory event with desaturation | |
| --- | --- | --- | --- | --- |
|  | **RR** | **QTc** | **RR** | **QTc** |
| **Exclusion of patients with less than 5 sequences (n=6)** | | | | |
| REM sleep | **-15.95**  **(-24.85, -7.05)** | 2.28  (0.35, 4.21) | **-17.11**  **(-24.16, -10.07)** | **3.71**  **(1.98, 5.45)** |
| Desaturation depth (%) | **-** | **-** | **-0.1**  **(-0.18, -0.02)** | **0.56**  **(0.41, 0.72)** |
| Arousal duration (s) | **-5.75**  **(-6.36, -5.15)** | **1.26**  **(1.13, 1.39)** | **-3.51**  **(-3.94, -3.09)** | **0.84**  **(0.74, 0.94)** |
| **Exclusion of patients with less than 10 sequences (n=15)** | | | | |
| REM sleep | **-15.81**  **(-24.87, -6.75)** | 2.19  (0.22, 4.15) | **-16.84**  **(-23.93, -9.75)** | **3.62**  **(1.88, 5.37)** |
| Desaturation depth (%) | **-** | **-** | **-0.1**  **(-0.18, -0.02)** | **0.57**  **(0.41, 0.72)** |
| Arousal duration (s) | **-5.68**  **(-6.29, -5.07)** | **1.25**  **(1.11, 1.38)** | **-3.51**  **(-3.93, -3.08)** | **0.84**  **(0.74, 0.95)** |
| **Removing sex variable from the model** | | |  |  |
| REM sleep | **-16.24**  **(-25.09, -7.38)** | 2.41  (0.48, 4.33) | **-17.27**  **(-24.3, -10.25)** | **3.81**  **(2.08, 5.54)** |
| Desaturation depth (%) | **-** | **-** | **-0.09**  **(-0.15, -0.03)** | **0.56**  **(0.4, 0.72)** |
| Arousal duration (s) | **-5.71**  **(-6.3, -5.11)** | **1.25**  **(1.12, 1.38)** | **-3.52**  **(-3.94, -3.09)** | **0.84**  **(0.74, 0.94)** |
| **Removing event type (apnea vs. hypopnea) from the model** | | | | |
| REM sleep | **-15.97**  **(-24.81, -7.13)** | 2.28  (0.36, 4.21) | **-17.61**  **(-24.66, -10.55)** | **3.83**  **(2.1, 5.57)** |
| Desaturation depth (%) | **-** | **-** | 0.27  (-0.34, 0.88) | **0.47**  **(0.32, 0.63)** |
| Arousal duration (s) | **-5.73**  **(-6.32, -5.14)** | **1.26**  **(1.13, 1.39)** | **-3.39**  **(-3.81, -2.97)** | **0.81**  **(0.71, 0.91)** |
| **Removing diagnosed hypertension variable from the model** | | | | |
| REM sleep | **-16.07**  **(-24.93, -7.21)** | 2.32  (0.4, 4.25) | **-17.17**  **(-24.21, -10.13)** | **3.72**  **(1.99, 5.45)** |
| Desaturation depth (%) | **-** | **-** | **-0.09**  **(-0.16, -0.02)** | **0.56**  **(0.4, 0.72)** |
| Arousal duration (s) | **-5.71**  **(-6.3, -5.11)** | **1.25**  **(1.12, 1.38)** | **-3.51**  **(-3.94, -3.09)** | **0.84**  **(0.74, 0.94)** |
| **Removing respiratory events duration variable from the model** | | | | |
| REM sleep | **-19.19**  **(-28.02, -10.37)** | **2.97**  **(1.05, 4.88)** | **-18.33**  **(-25.36, -11.3)** | **4.17**  **(2.43, 5.91)** |
| Desaturation depth (%) | **-** | **-** | **-0.42**  **(-0.72, -0.12)** | **0.7**  **(0.54, 0.85)** |
| Arousal duration (s) | **-5.79**  **(-6.39, -5.19)** | **1.27**  **(1.14, 1.4)** | **-3.58**  **(-4, -3.16)** | **0.87**  **(0.76, 0.97)** |

Mixed effect models were adjusted for age, sex, body mass index, sleep-stage (NREM vs. REM), pre-event values, and diagnosed hypertension, with patients as the random effect. Model estimates are presented as raw values in milliseconds. The *p*-values are adjusted with Bonferroni correction. Bold typeface font denotes *p* < 0.05. QTc = heart rate corrected QT interval, BMI = body mass index, REM = rapid eye movement sleep.
